# Dichotomous factor analysis of symptoms reported by UK and US veterans of the 1991 Gulf War

**DOI:** 10.1186/1478-7954-2-8

**Published:** 2004-09-03

**Authors:** Rosane Nisenbaum, Khalida Ismail, Simon Wessely, Catherine Unwin, Lisa Hull, William C Reeves

**Affiliations:** 1 Division of Viral and Rickettsial Diseases, National Center for Infectious Diseases, Centers for Disease Control and Prevention, Atlanta, GA 30333 USA; 2 Gulf War Illness Research Unit, Guy's, King's, and St Thomas's Medical School, London UK

## Abstract

**Background:**

Factor analysis is one of the most used statistical techniques to analyze the inter-relationships among symptoms reported by Gulf War veterans. The objective of this study was to apply factor analyses to binary symptom data from the UK study of Gulf War illness and the US Air Force study of Gulf War veterans, and to compare the symptom domains derived from the distinct samples.

**Methods:**

UK veterans of the 1991 Gulf War (n = 3,454), individuals deployed to Bosnia on U.N. peacekeeping operations (n = 1,979) and Gulf War-era servicemen (n = 2,577) who were not deployed to the Gulf were surveyed in 1997–1998, and US 1991 Gulf War veterans from four Air Force units (n = 1,163) were surveyed in 1995 to collect health characteristics including symptoms. Each sample was randomly split in half for exploratory and confirmatory dichotomous factor analyses with promax oblique rotation.

**Results:**

Four correlated factors were identified in each of the samples. Three factors (Respiratory, Mood-Cognition, Peripheral Nervous) overlapped considerably across the UK cohorts. The Gastrointestinal/Urogenital factor in the UK Gulf cohort was noticeably different from the Gastrointestinal factor identified from the Bosnia and Era cohorts. Symptoms from Gulf War UK and U.S cohorts yielded similar Gastrointestinal, Respiratory and Mood-Cognition factors, despite differences in symptom inventories between the two surveys. A Musculoskeletal factor was only elicited from the US Gulf sample.

**Conclusion:**

Findings of this report are consistent with those from other factor analysis studies that identified similar symptom dimensions between Gulf and non-Gulf War veterans, except that the Gastrointestinal factor in Gulf veterans included other symptom types. Correlations among factors raise the question as to whether there is a general illness, even if not unique to Gulf veterans, representing the common pathway underlying the identified factors. Hierarchical factor analysis models may be useful to address this issue.

## Introduction

Reports that veterans of the 1991 Gulf War were suffering from unexplained signs and symptoms started to appear as early as one year after the conflict [1]. Veterans complained of several symptoms including myalgia, arthralgia and debilitating fatigue, but no cause could be found. Over a decade later, it remains uncertain whether or not Gulf War illness is a specific response to a specific exposure/hazard, or alternatively a non-specific response to what may be a variety of hazards/stressors and circumstances [2]. Factor analysis may assist this debate by determining whether or not there is a specific structure to the symptoms endorsed by Gulf War veterans that differentiates them from symptoms shown by non-Gulf veterans.

Several studies have applied factor analysis (or principal components analysis) to examine and compare the inter-relationships among symptoms reported by veterans [3-13]. In general, factor structures have been found to be similar in veterans deployed and not deployed to the Gulf War [3,9-11]. Despite different symptom inventories, differences in analytical procedures, and personal choices for factor labeling, studies of Gulf War illness report between three and seven factors that represent combinations of the following domains: (a) mood, cognition, fatigue, psychological; (b) respiratory condition; (c) neurological condition; (d) musculoskeletal pain; (e) peripheral nervous system; (f) gastrointestinal disorder; and (g) mixed somatic complaints.

When symptoms are measured on a continuous scale normally distributed, linear factor analysis (e.g. common factor analysis [14]) can be applied. However, when symptoms are measured on a nominal (Yes/No) or binary scale (0/1), linear factor analysis may yield biased estimates of the factor structure [15,16]. A dichotomous factor analysis model [15] would be more appropriate.

The objective of this report was to apply dichotomous factor analyses to binary symptom data from two studies, i.e., the UK study of Gulf War illness [11,17-19] and the US Air Force study of Gulf War veterans [12], and to assess whether observed symptom patterns represented similar syndromes across nations.

## Methods

### Sources of data

The data used in this report came from two sources: the UK Study of Gulf War illness [11,17-19] that included three cohorts: individuals deployed to the Persian Gulf in 1991 or to Bosnia on U.N. peacekeeping operation, and Gulf War-era servicemen who were not deployed to the Gulf, and a study of US Air Force Gulf War veterans [12]. Briefly, the UK study was a postal survey conducted between August 1997 and November 1998 that asked veterans of the Royal Navy, Army, Royal Air Force about socio-demographic, military and health characteristics [17-19]. In Gulf veterans, previous work has shown that ill health was associated with rank and socio-demographic factors but it was similar across all three services [19]. Also, linear factor analysis and cluster analysis indicated that symptom patterns were similar across the three cohorts [11,17]. In the US study, four Air Force units (2 in Pennsylvania and 2 in Florida) were surveyed between January and March 1995. Questionnaires were distributed to volunteers and queried about deployment to the Persian Gulf, health status, demographic and military characteristics and symptoms [12]. A case definition of multi-symptom illness was derived using linear factor analysis [12].

### Symptoms

In the UK study, we considered the analysis of 50 symptoms that occurred in the preceding month ("During the past month, have you suffered from any of the following symptoms?") [11,17,18] (Table 1). In the US study, we considered 35 symptoms that were reported as current health problems [12] (Table 2). The analyses only included subjects with complete symptom data (i.e., only a small proportion of veterans had missing symptoms: 2.2% in the UK Gulf cohort, 3.5% in the UK Bosnia cohort, and 1.4% in the UK Era cohort, 0% in the US study).

**Table 1:** Prevalence (%) of symptoms present in the past month across UK Study: Gulf, Bosnia and Era Cohorts

During the past month have you suffered from:	Gulf* N = 3,454	Bosnia N = 1,979	Era N = 2,577
Feeling unrefreshed after sleep	56.1	32.5	31.5
Irritability/outburst of anger	54.7	32.2†	25.5
Headaches	54.2	36.5	36.8
Fatigue	51.1	26.9	28.2
Sleeping difficulties	47.8	30.5	28.3
Forgetfulness	44.7	19.4†	16.8
Loss of concentration	39.5	16.6	15.0
Joint stiffness	39.3	20.9	22.8
Flatulence or burping	34.0	15.7†	21.0
Pain without swelling or redness in several joints	31.7	13.7	14.2
Feeling distant or cut off from others	27.9	14.4†	10.3
Avoiding doing things/situations	26.5	12.4†	10.1
Feeling jumpy/easily startled	24.7	12.9†	9.4
Chest pain	24.5	12.5	11.6
Tingling in fingers and arms	24.3	8.4†	10.9
Night sweats that soak the bed sheets	23.7	12.0†	9.5
Itchy or painful eyes	22.9	10.5	11.9
Sore throat	22.3	15.1	13.6
Distressing dreams	21.6	13.1†	9.0
Numbness or tingling in fingers or toes	21.4	8.1†	10.9
Ringing in the ears	20.5	10.8	12.5
Wheezing	20.4	10.1	9.7
Diarrhea	20.2	11.1	11.9
Unable to breathe deeply enough	20.0	9.8†	7.8
Unintended weight gain greater than 10 lbs	18.7	10.8†	8.5
Dry mouth	17.4	9.1†	6.6
Loss of interest in sex	17.3	7.1	6.7
Dizziness	17.0	7.0	7.7
Tingling in legs and arms	16.8	5.4†	6.8
Rapid heartbeat	16.4	7.5	7.6
Feeling short of breath at rest	15.3	6.5	5.5
Increased sensitivity to noise	15.0	6.3	5.6
Increased sensitivity to light	14.7	6.0	5.8
Stomach cramp	14.6	7.8	7.5
Passing urine more often	14.3	4.9†	6.3
Persistent cough	13.9	7.8†	5.8
Loss or decrease in appetite	13.3	8.5†	5.2
Intolerance to alcohol	11.9	5.0	4.0
Shaking	11.6	5.3†	3.7
Constipation	10.9	5.9	5.2
Faster breathing than normal	10.4	4.4	3.3
Feeling disoriented	10.3	3.2	3.5
Feeling feverish	8.7	3.5	3.0
Nausea	8.7	3.7	3.7
Lump in throat	8.0	3.8	3.0
Unintended weight loss greater than 10 lbs.	5.5	3.9†	2.6
Double vision	5.4	2.5	2.1
Pain on passing urine	5.2	2.3	1.9
Burning sensation in sex organs	5.0	1.3	1.6
Vomiting	4.7	3.2	2.8
In general would you say your health is (mean, standard deviation) 1=Excellent, 2=Very Good, 3=Good, 4=Fair, 5=Poor	2.8 (1.1)‡	2.3 (1.0)	2.3 (1.0)

* Gulf War veterans significantly different (p < 0.05) from Era and Bosnia veterans with respect to all symptoms

† Bosnia veterans were significantly different (p < 0.05) from Era veterans.

‡ Gulf War veterans significantly different (p < 0.05) from Bosnia and Era veterans

**Table 2:** Prevalence (%) of symptoms reported as current health problems in the US Study of Gulf War veterans

Current health problem	Deployed to the Gulf (N = 1,163)
Sinus congestion	51.8
Headache	50.0
Fatigue	42.9
Joint pain	35.5
Difficulty remembering or concentrating	34.4
Joint stiffness	30.4
Difficulty or problems to sleep	27.6
Gas, bloating, cramps or abdominal pain	26.7
Trouble finding words	26.1
Irritability or moodiness	25.5
Skin rashes or sores	23.0
Numbness or tingling in fingers or toes	21.0
Muscle pains	19.9
Hay fever or other allergies	19.0
Depression	18.0
Diarrhea (3 or more loose bowel movements in 24 hours)	17.6
Sore throat	17.4
Cough	17.1
Anxiety	17.0
Unintended weight gain greater than 10 lbs	16.9
Shortness of breath	16.4
Chest pain	15.0
Decreased sexual interest	14.3
Dizziness	13.9
Night sweats that soak your bed sheets	13.3
Fatigue lasting 24 hours after exertion	12.6
Sores inside your nose	10.8
Swollen lymph glands in your neck, armpit, groin	10.1
Inability to tolerate milk	7.1
Episodes of disorientation	6.6
Nausea or vomiting	6.3
Wheezing	5.9
Sensitivity to chemicals	5.2
Fever	4.9
Unintended weight loss greater than 10 lbs	2.7
In general would you say your health is (mean, standard deviation) 1=Excellent, 2=Very Good, 3=Good, 4=Fair, 5=Poor	2.3 (0.9)

### Statistical analyses

We used chi-squared tests to compare symptom reporting among the UK Gulf, Bosnia and non-deployed cohorts, and between the UK and US Gulf War veterans. For factor analyses, data from each UK group and US study were randomly split into 2 halves (exploratory and confirmatory samples). Exploratory dichotomous factor analyses [20] were performed to determine the number of factors that explained the correlations among symptoms. Confirmatory dichotomous factor analyses [20] were conducted to test the reproducibility of the factor structure identified in the exploratory phase. We used a robust weighted least squares estimator to calculate factor loadings for the dichotomous model [20]. The promax oblique rotation was used to estimate factor correlations. For exploratory analyses, the scree plot was used to estimate the number of factors and utilized eigenvalues from the tetrachoric correlation matrix. The number of factors was considered sufficient to explain symptom correlations if the root mean square error of approximation (RMSEA) was ≤ 0.06 [20,21]. Since in general, factor loadings are considered meaningful when they exceed 0.30 or 0.40 [14], we determined the stability of the factor structures by repeating the exploratory factor analyses in the exploratory sample after eliminating symptoms with factor loadings of <0.40.

The confirmatory dichotomous model [20] specified the number of factors and the leading symptom in each factor (i.e., one with highest loading in its factor, and fixed zero loadings in the remaining factors) to test the exploratory structure in the confirmatory sample. We also set the factor variances to 1 so that the model would be identifiable. No other parameters were fixed. The confirmatory model was deemed to fit the data well if any of the following goodness-of-fit indices was satisfied: RMSEA of ≤ 0.06, Tucker-Lewis Index (TLI) of ≥ 0.95, Comparative Fit Index (CFI) of ≥ 0.95, or standardized root mean square residual (SRMR) of ≤ 0.08 [21,22]. Finally, we fitted the confirmatory model to data from all subjects from the exploratory and confirmatory samples. We used M-plus version 2.14 [20] to fit the dichotomous factor models and SAS version 8.1 (SAS Inc., Cary, NC) to perform all other analyses.

## Results

### Description of the samples

#### The UK Study

Of the 3,454 veterans of the Gulf War, 93.3% were men, 75% were married or living with a partner, 92.5% had regular military status when they were deployed to the Gulf. Their average age was 34.4 years (standard deviation = 6.8). The Bosnia cohort had 1,979 veterans with average age of 29.3 years (standard deviation = 6.7) including 89.4% men, 57.5% married or living with a partner, and 91.1% with regular military status when deployed to Bosnia. The Era cohort included 2,577 veterans (92.7% men, 75.3% married or living with a partner, average age of 35.3 years (standard deviation = 7.2) and 48.8% regular military status).

#### The US Air Force Study

The US Gulf sample included 1,163 veterans who were 94.0% men, 74.9% married or living with a partner, with an average age of 37.9 years (standard deviation = 8.4).

### Symptom distribution

The most common symptoms across all UK groups were feeling unrefreshed after sleep, irritability, headaches, fatigue, sleeping difficulties, forgetfulness, loss of concentration, joint stiffness and flatulence/burping (Table 1). The prevalence of all symptoms reported by Gulf War veterans was significantly higher than that reported by Bosnia or Era veterans. On average, veterans of all groups reported their general health was at least good. However, scores for Gulf War veterans were significantly lower than those for Bosnia or Era veterans (t-test with Bonferroni adjustment, p-value <0.05). Table 2 displays the symptom distribution among US veterans of the Gulf War and Table 3 shows the equivalence between symptoms assessed in the UK and US studies. Sixteen symptoms in the UK study were not assessed in the US study, and 11 symptoms in the US study were not assessed in the UK study. Among the 24 symptoms that were equivalent in both studies, 18 were significantly more prevalent among UK Gulf veterans than their US counterparts (chi-square p-value <0.05).

**Table 3:** Equivalence between symptoms in UK and US Studies*

**UK Study**	**US Study**
Irritability/outburst of anger	Irritability or moodiness
Headaches	Headaches
Fatigue	Fatigue
Sleeping difficulties	Difficulty or problems to sleep
Forgetfulness OR Loss of concentration	Difficulty remembering or concentrating
Joint stiffness	Joint stiffness
Flatulence or burping OR Stomach cramp	Gas, bloating, cramps or abdominal pain
Pain without swelling or redness in several joints	Joint pain
Chest pain	Chest pain
Night sweats that soak the bed sheets	Night sweats that soak your bed sheets
Sore throat	Sore throat
Numbness or tingling in fingers or toes	Numbness or tingling in fingers or toes
Wheezing	Wheezing
Diarrhea	Diarrhea (3 or more loose bowel movements in 24 hours)
Unintended weight gain greater than 10 lbs	Unintended weight gain greater than 10 lbs
Loss of interest in sex	Decreased sexual interest
Dizziness	Dizziness
Feeling short of breath at rest	Shortness of breath
Persistent cough	Cough
Feeling disoriented	Episodes of disorientation
Feeling feverish	Fever
Nausea OR Vomiting	Nausea or Vomiting
Unintended weight loss greater than 10 lbs.	Unintended weight loss greater than 10 lbs.
Diagnostic Criteria for Anxiety Disorder† Feeling distant or cut off from others OR Feeling jumpy/easily startled OR Unable to breathe deeply enough OR Tingling in legs and arms OR Rapid heartbeat OR Shaking OR Faster breathing than normal OR Lump in throat	Anxiety
**16 could not find equivalent: **Avoiding doing things/situations, Tingling in fingers and arms, Feeling unrefreshed after sleep, Itchy or painful eyes, Distressing dreams, Ringing in the ears, Dry mouth, Increased sensitivity to noise, Increased sensitivity to light, Passing urine more often, Loss or decrease in appetite, Intolerance to alcohol, Constipation, Double vision, Pain on passing urine, Burning sensation in sex organs	**11 could not find equivalent: **Trouble finding words, Depression, Muscle pains, Hay fever or other allergies, Fatigue lasting 24 hrs after exertion, Swollen lymph glands in your neck, armpit, groin, Inability to tolerate milk, Sensitivity to chemicals, Sinus congestion, Skin rashes or sores, Sores inside your nose

†All equivalent symptoms were significantly more prevalent among UK Gulf veterans than among UK Gulf veterans, except cough and joint pain that were less prevalent, and diarrhea, numbness or tingling in fingers or toes, shortness of breath, and weight gain that were not different in the 2 samples.

†*Diagnostic and statistical manual of mental disorders: DSM-IV–4th ed. *Washington, DC: American Psychiatric Association; 1994

### Dichotomous Factor Analyses

#### UK Gulf Cohort

The exploratory sample consisted of 1,783 persons. The scree plot suggested 1 major factor, but it was not clear how many additional factors should be investigated (data available from authors). We removed the first eigenvalue from the plot, to better determine how much each additional factor contributed to the variance, and decided to examine 2 to 5-factor solutions. Although all solutions indicated a good fit between data and model (RMSEA ≤ 0.06), the 2-, and 3- factor models yielded non-interpretable factors, and the 5-factor solution could not be confirmed. Thus, we tested the 4-factor exploratory solution in the confirmatory sample that included 1,671 subjects. We specified the number of factors to be 4, the factor variances to be 1 and the leading symptoms of the first (pain on passing urine), second (loss of concentration), third (unable to breathe deeply enough), and fourth (tingling in fingers and arms) factors. Table 4 summarizes the final confirmatory 4-factor solution using data from all 3,454 subjects and 35 symptoms. This model fits the data well (CFI = 0.95, TLI = 0.98, RMSEA = 0.04, SRMR = 0.05). The order of the factors is not important, because in this model the variances were standardized to 1. The factors were labeled Gastrointestinal/Urogenital, Respiratory, Mood-Cognition, and Peripheral Nervous. The Respiratory, Mood-Cognition and Peripheral Nervous factors represent the same domains as the three factors in the study by Ismail et al [11]. This solution also yielded a very high correlation among the factors (range = 0.52–0.62). We could not estimate the variance explained by each factor, because the model standardized all variances to 1. Since factors correlate in an oblique solution, it is quite complex to calculate the proportion of variance explained by each factor. However, for descriptive purposes, we used the varimax orthogonal solution of the exploratory factor model to have an idea of the importance of each factor. Using all 3,454 subjects, 35 symptoms, and the 4-factor exploratory model we estimated that the proportion of variance explained by each factor was 16.3% (Gastrointestinal/Urogenital), 10.1% (Respiratory), 22.4% (Mood-Cognition), and 8% (Peripheral Nervous). Thus the Mood-Cognition Factor contributes most of the variance in the data, followed by the Gastrointestinal/Urogenital Factor.

**Table 4:** Factor loadings for final 4-factor confirmatory model for symptoms reported in the UK Study-Gulf Cohort (N = 3,454), UK Study-Bosnia Cohort (N = 1,979), and UK Study-Era Cohort (N = 2,577)

During the past month have you suffered from:	**Gastrointestinal/Urogenital**	**Gastrointestinal**		
	**Gulf**	**Bosnia**	**Era**
Nausea	**.78**	**.56**	**-**
Vomiting	**.77**	-	-
Diarrhea	**.76**	**.76**	**.76**
Stomach cramp	**.74**	**.73**	**.71**
Constipation	**.59**	**.78**	**.57**
Flatulence or burping	**.55**	**.63**	**.58**
Sore throat	**.53**	**.49**	-
Feeling feverish	**.52**	**.51**	-
Dry mouth	**.48**	**.49**	-
Pain on passing urine	**.53**	-	-
Burning sensation in sex organs	**.48**	-	-
Headaches	**.48**	-	-
Loss or decrease in appetite	**.47**	-	-
Unintended weight loss greater than 10 lbs.	**.43**	-	-
	**Respiratory**		
	**Gulf**	**Bosnia**	**Era**
Unable to breathe deeply enough	**.89**	**.87**	**.89**
Wheezing	**.77**	**.75**	**.91**
Feeling short of breath at rest	**.73**	**.89**	**.81**
Faster breathing than normal	**.66**	**.63**	**.66**
Persistent cough	**.50**	-	**.48**
Chest pain	-	**.61**	-
Rapid heartbeat	-	**.51**	-
	**Mood-Cognition**		
	**Gulf**	**Bosnia**	**Era**
Loss of concentration	**.93**	**.70**	**.79**
Forgetfulness	**.87**	**.66**	**.71**
Feeling distant or cut off from others	**.77**	**.80**	**.86**
Avoiding doing things/situations	**.74**	**.67**	**.75**
Irritability/outburst of anger	**.64**	**.71**	**.74**
Feeling unrefreshed after sleep	**.62**	**.68**	-
Feeling jumpy/easily startled	**.60**	**.73**	**.79**
Sleeping difficulties	**.57**	**.66**	**.56**
Feeling disoriented	**.57**	**.64**	**.70**
Fatigue	**.57**	**.53**	-
Increased sensitivity to noise	**.56**	**.58**	**.63**
Distressing dreams	**.52**	**.66**	**.78**
Loss of interest in sex	**.51**	**.54**	**.65**
Intolerance to alcohol	-	**.50**	**.46**
Shaking	-	**.45**	**.55**
Night sweats	-	-	**.52**
	**Peripheral Nervous**		
	**Gulf**	**Bosnia**	**Era**
Tingling in fingers and arms	**.97**	**.99**	**.91**
Numbness or tingling in fingers or toes	**.84**	**.89**	**.90**
Tingling in legs and arms	**.77**	**.80**	**.89**

- Blank entries in the table indicate that symptom had a factor loading < .40 during the exploratory phase and was not considered in the confirmatory model

#### UK Bosnia Cohort

The Bosnia Cohort exploratory sample included 1,008 subjects and the confirmatory included 971. A 4-factor solution with 32 symptoms was confirmed (goodness-of-fit measures: CFI = 0.961; TLI = 0.986; RMSEA = 0.029; SRMR = 0.045; factor correlations range = 0.44–0.63). Results for all 1,979 subjects are displayed in Table 4. The proportion of variance explained by each factor, based on the orthogonal varimax solution was 13.4% (Respiratory), 24.7% (Mood-Cognition), 9.4% (Peripheral Nervous), and 14.6% (Gastrointestinal).

Although the constructs identified from symptoms reported by Bosnia veterans were similar to those confirmed in the Gulf War veteran sample, we were unable to confirm the Gulf War confirmatory 4-factor solution in the Bosnia sample. Some symptoms from the Gulf cohort Gastrointestinal/Urogenital factor loaded in several Bosnia cohort factors, yielding a structure that was difficult to interpret.

#### UK Era Cohort

There were 1,325 observations in the exploratory and 1,252 in the confirmatory samples. A confirmatory 4-factor model with 26 symptoms and all 2,577 subjects is displayed in Table 4 (goodness-of-fit measures: CFI = 0.99, TLI = 1.00, RMSEA = 0.02, SRMR = 0.04; factor correlations range = 0.41–0.58). Fatigue and unrefreshing sleep could not be confirmed in any factor. The proportion of variance explained by each factor based on the orthogonal varimax exploratory solution was 13.3% (Respiratory), 28.8% (Mood-Cognition), 10.3% (Peripheral Nervous), and 9.9% (Gastrointestinal).

### Comparing the factor structures of the UK Gulf Cohort with Bosnia and Era Cohorts

The overlap of symptom composition across samples was remarkable for the Respiratory, Peripheral Nervous and Mood-Cognition factors (Table 4). The factor mostly defined by gastrointestinal symptoms comprised the major difference among the cohorts.

#### US Air Force Gulf War Study

The exploratory sample consisted of 590 subjects and the confirmatory included 573. A 4-factor solution with 26 symptoms was confirmed and results for all 1,163 subjects are displayed in Table 5. The proportion of variance explained by each factor based on the orthogonal varimax exploratory solution was 19.1% (Gastrointestinal/Respiratory), 7.7% (Allergies), 20.7% (Mood-Cognition), and 10.8% (Musculoskeletal).

**Table 5:** Factor loadings for final confirmatory 4-factor model for symptoms reported in the US Study of Gulf War veterans (N = 1,163)

Current health problem	Factor 1	Factor 2	Factor 3	Factor 4
	**Gastrointestinal/ Respiratory**	**Allergies**	**Mood-Cognition**	**Musculoskeletal**

Nausea or vomiting	**.80**	.00	.00	.00
Diarrhea (3 or more loose bowel movements in 24 hours)	**.73**	.06	-.04	.02
Gas, bloating, cramps or abdominal pain	**.71**	-.01	.06	.02
Skin rashes or sores	**.56**	-.10	-.05	.20
Sore throat	**.54**	.36	-.17	.14
Fever	**.53**	.18	.06	.17
Swollen lymph nodes in your neck, armpit, groin	**.52**	.23	-.14	.08
Night sweats that soak your bed sheets	**.52**	-.07	.10	.16
Wheezing	**.52**	. 22	-.10	.18
Cough	**.47**	.36	-.16	.13
Chest pain	**.44**	.16	.13	.12
Sinus congestion	.00	**.90**	.00	.00
Hay fever or other allergies	-.04	**.60**	.05	-.09
Difficulty remembering or concentrating	.00	.00	**.87**	.00
Depression	-.07	.17	**.77**	-.08
Trouble finding words	. 04	.05	**.75**	-.11
Irritability or moodiness	-.11	.28	**.74**	.00
Anxiety	-.05	.29	**.73**	-.12
Episodes of disorientation	.25	-.01	**.66**	-.03
Fatigue lasting 24 hours after exertion	.15	.05	**.48**	.28
Fatigue	.15	.13	**.48**	.24
Decreased sexual interest	.15	.00	**.45**	.07
Dizziness	.36	.04	**.40**	.01
Joint pain	.00	.00	.00	**.96**
Joint stiffness	.06	.01	.08	**.80**
Muscle pain	.07	.03	.15	**.65**

Goodness-of-fit measures: CFI = .970; TLI = .988; RMSEA = .029; SRMR = .043

Inter-factor correlations: Factor1, Factor2 = .474; Factor1, Factor3 = .670; Factor1, Factor4 = .525; Factor2, Factor3 = .388;

Factor2, Factor4 = .488; Factor3, Factor4 = .511

### Comparing the factor structures of the UK Gulf Cohort with US Gulf Cohort

We could not directly compare the factor structures because the symptom inventories were so different. For example, 3 peripheral nervous symptoms were asked from the UK veterans (Tingling in fingers and arms, Numbness or tingling in fingers or toes, Tingling in legs and arms) while the US study included only 1 (numbness or tingling in fingers or toes). Thus, a separate factor could not be derived from the US study. Nevertheless, both UK and US data yielded similar constructs, namely a mixed gastrointestinal factor, a mood-cognition factor, and a respiratory-related factor. The main difference was that the musculoskeletal construct could not be confirmed in the UK Gulf cohort, and it represented a separate factor in the US sample (Tables 4 and 5).

## Discussion

The objective of this report was to identify and compare syndromes among 4 samples collected from UK [11,17-19] and US [11] studies of Gulf War illness by using factor analysis. We used dichotomous factor analysis models because symptoms were measured on a nominal scale (Yes/No), either during the past month (UK study) or currently (US study). UK data included Gulf War servicemen, individuals deployed to Bosnia on a U.N. peacekeeping operation, and active duty military that had not been deployed. US data included only Gulf War veterans.

We identified and confirmed at most 4 correlated factors in each of the samples. Three of the four constructs (Respiratory, Mood-Cognition, Peripheral Nervous) overlapped considerably across the UK cohorts. These factors were identical to those derived in a linear factor analysis of these data [11]. However, the current study identified one factor including gastrointestinal and urogenital symptoms in the UK Gulf cohort that was noticeably different from the gastrointestinal factor identified from the Bosnia and Era cohorts. One possible explanation is that Gulf War veterans were more stressed than Bosnia or Era veterans, and this fact maybe associated with multi-system symptom reporting. More needs to be investigated in this area.

In addition, despite differences in study designs, methods of data collection, military populations and symptom inventories between the UK and US studies of Gulf War veterans, Gastrointestinal, Respiratory and Mood-cognition factors were identified in both UK and US studies. Of note, although joint pain and joint stiffness were measured in both UK and US samples of Gulf War veterans, a Musculoskeletal factor was only elicited as a separate factor from the US data.

In general, findings of this report were consistent with those from other studies that used factor analysis of symptoms to compare symptom patterns between Gulf and non-Gulf War veterans [3,9-11]. However, in one comparative study, the factor structure derived from symptoms reported by Gulf War veterans included a neurological impairment factor that was absent among non-Gulf War subjects [6]. Based on our experience in analyzing symptom data, we suggest that, to achieve more precise comparability across studies, a standardized symptom questionnaire be developed and used on future studies of war-related illnesses. For example, symptoms can be measured on an interval, rather than binary or nominal scale, accounting for frequency and intensity as in the Psychosomatic Symptom Checklist [23].

In this report, we encountered difficulties confirming the dichotomous factor structures, or reproducing a factor structure in another sample, because many symptoms were rare, which created numerical problems. On the other hand, failure to reproduce factor structures across samples may also be due to different symptom distributions in the samples being considered, as was the case of the UK Gulf and Bosnia cohorts. We also acknowledge the difficulties in analyzing self-report symptom data. However, since the UK and US studies were independent of the military and confidential, we do not believe there was a reason form service personnel to exaggerate symptoms in order to gain compensation or eligibility for veterans.

Finally, it must be noted that, in each of the UK or US cohorts, factors were moderately or highly correlated. Correlated factors are complex to interpret because it is difficult to separate their independent effects [24]. This finding raises the question as to whether there is higher-order dimension, or general illness, representing the common pathway underlying all four factors. Hierarchical factor analysis models [24-26] may be useful in addressing this issue.

In conclusion, considerable progress has been made in defining medically unexplained illness associated with deployment to the 1991 Gulf War. Our results from independent studies conducted in the UK and US confirmed occurrence of an illness comprised of 4 correlated groups of symptoms (factors) in deployed military personnel from both countries. Similar illness occurred in troops who did not participate in the Gulf War (albeit at lower rates and with different specific characteristics), so we believe that this pattern of symptoms is not unique to Gulf War service nor does it represent a unique illness or "Gulf War syndrome." In fact, similar illnesses to those affecting Gulf War veterans have been noted among veterans of US Civil War [27] and British Boer War [28]. Similar illnesses can also be expected to occur in association with current deployments in Afghanistan and Iraq. A better understanding of predisposing, precipitating, and perpetuating factors must be obtained to provide appropriate care for veterans and to devise prevention strategies. A central question remains: how to resolve whether such illnesses reflect a common pathophysiologic process.

## Competing Interests

None declared.

## Authors' Contributions

RN conceived of this analysis was responsible for its execution and had primary responsibility for the manuscript; KI was instrumental in the conception and design of the UK veterans' study and had primary responsibility for its analysis; collaborated in analysis and interpretation of the present data and writing the manuscript; SW was Principal Investigator for the UK veterans' study, Collaborated in the concept of the present study and collaborated in analysis, interpretation and the manuscript; CU collaborated in the UK veteran's study and collaborated in analysis and interpretation of data and drafting the manuscript for this study; LH collaborated in the UK veteran's study and collaborated in analysis and interpretation of data and drafting the manuscript for this study; WCR was Principal Investigator of the US Gulf War study, conceived the idea for the present study, served as Principal Investigator for the present study and collaborated in all aspects of data interpretation and writing the manuscript.
